# Basic level scene understanding: categories, attributes and structures

**DOI:** 10.3389/fpsyg.2013.00506

**Published:** 2013-08-29

**Authors:** Jianxiong Xiao, James Hays, Bryan C. Russell, Genevieve Patterson, Krista A. Ehinger, Antonio Torralba, Aude Oliva

**Affiliations:** ^1^Computer Science, Princeton UniversityPrinceton, NJ, USA; ^2^Computer Science, Brown UniversityProvidence, RI, USA; ^3^Computer Science and Engineering, University of WashingtonSeattle, WA, USA; ^4^Brain and Cognitive Sciences, Massachusetts Institute of TechnologyCambridge, MA, USA; ^5^Department of EECS, Computer Science and Artificial Intelligence Laboratory, Massachusetts Institute of TechnologyCambridge, MA, USA; ^6^Computer Science and Artificial Intelligence Laboratory, Massachusetts Institute of TechnologyCambridge, MA, USA

**Keywords:** SUN database, basic level scene understanding, scene recognition, scene attributes, geometry recognition, 3D context

## Abstract

A longstanding goal of computer vision is to build a system that can automatically understand a 3D scene from a single image. This requires extracting semantic concepts and 3D information from 2D images which can depict an enormous variety of environments that comprise our visual world. This paper summarizes our recent efforts toward these goals. First, we describe the richly annotated SUN database which is a collection of annotated images spanning 908 different scene categories with object, attribute, and geometric labels for many scenes. This database allows us to systematically study the space of scenes and to establish a benchmark for scene and object recognition. We augment the categorical SUN database with 102 scene attributes for every image and explore attribute recognition. Finally, we present an integrated system to extract the 3D structure of the scene and objects depicted in an image.

## 1. Introduction

The ability to understand a 3D scene depicted in a static 2D image goes to the very heart of the computer vision problem. By “scene” we mean a place in which a human can act within or navigate. What does it mean to *understand a scene*? There is no universal answer as it heavily depends on the task involved, and this seemingly simple question hides a lot of complexity.

The dominant view in the current computer vision literature is to name the scene and objects present in an image. However, this level of understanding is rather superficial. If we can reason about a larger variety of semantic properties and structures of scenes it will enable richer applications. Furthermore, working on an over-simplified task may distract us from exploiting the natural structures of the problem (e.g., relationships between objects and 3d surfaces or the relationship between scene attributes and object presence), which may be critical for a complete scene understanding solution.

What is the ultimate goal of computational scene understanding? One goal might be to pass the **Turing test for scene understanding**: Given an image depicting a static scene, a human judge will ask a human or a machine questions about the picture. If the judge cannot reliably tell the machine from the human, the machine is said to have passed the test. This task is beyond the current state-of-the-art as humans could ask a huge variety of meaningful visual questions about an image, e.g., Is it safe to cross this road? Who ate the last cupcake? Is this a fun place to vacation? Are these people frustrated? Where can I set these groceries? etc.

Therefore, we propose a set of goals that are suitable for the current state of research in computer vision that are not too simplistic nor challenging and lead to a natural representation of scenes. Based on these considerations, we define the task of scene understanding as predicting the scene category, scene attributes, the 3D enclosure of the space, and all the objects in the images. For each object, we want to know its category and 3D bounding box, as well as its 3D orientation relative to the scene. As an image is a viewer-centric observation of the space, we also want to recover the camera parameters, such as observer viewpoint and field of view. We call this task **basic level scene understanding**, with analogy to basic level in cognitive categorization (Rosch, 1978). It has practical applications for providing sufficient information for simple interaction with the scene, such as navigation and object manipulation.

### 1.1. Outline

In this paper we discuss several aspects of basic level scene understanding. First, we quickly review our recent work on categorical (section 2) and attribute-based scene representations (section 3). Finally, we go into greater detail about novel work in 3d scene understanding using structured learning to simultaneously reason about many aspects of scenes (section 4).

**Figure 1:**
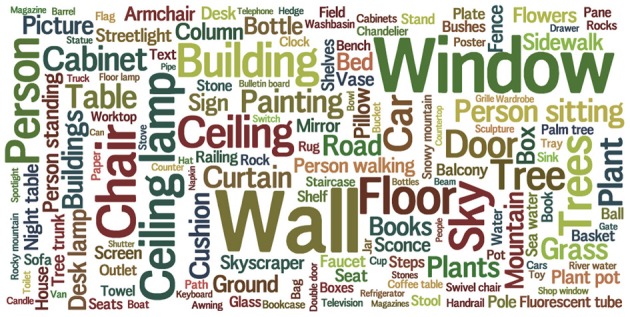
**Object categories in the SUN database**. The area of each word is proportional to the frequency of that object category.

**Figure 2:**
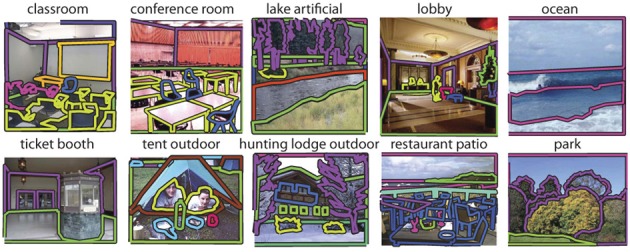
**Examples from the 19,503 fully annotated images in the SUN database**.

**Figure 3:**
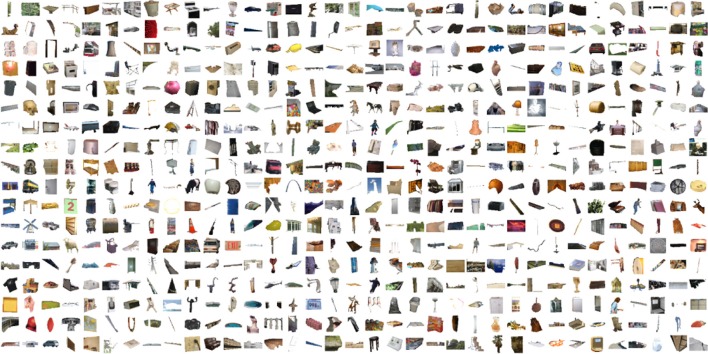
**Sample object segments from popular object categories in the SUN database**.

**Figure 4:**
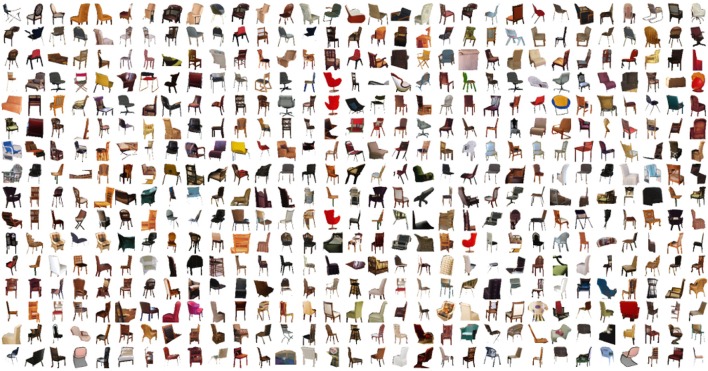
**To demonstrate intra-category object variation within the SUN database, these are samples of the 12,839 chairs that were manually annotated in 3500 images**.

Supporting these research efforts is the **S**cene **UN**derstanding (SUN) database. By modern standards, the SUN database is not especially large, containing on the order of 100,000 scenes. But the SUN database is, instead, **richly annotated** with scene categories, scene attributes, geometric properties, “memorability” measurements (Isola et al., 2011), and object segmentations. There are 326,582 manually segmented objects for the 5650 object categories labeled (Barriuso and Torralba, 2012). Object categories are visualized in Figure 1 and annotated objects are shown in Figures 2, 3, and 4. We believe the SUN database is the largest database from which one can learn the relationship among these object and scene properties. This combination of scene diversity and rich annotation is important for scaling scene understanding algorithms to work in the real world.

## 2. Scene categories

One of the fundamental tasks of basic level scene understanding is to be able to classify a natural image into a limited number of semantic categories. What are the scene categories? From a human-centric perspective, the categories should capture the richness and diversity of environments that make up our daily experiences. Although the visual world is continuous, many environmental scenes are visual entities that can be organized in functional and semantic groups. A given scene or place may allow for specific actions, such as eating in a restaurant, drinking in a pub, reading in a library, or sleeping in a bedroom.

**Figure 5:**
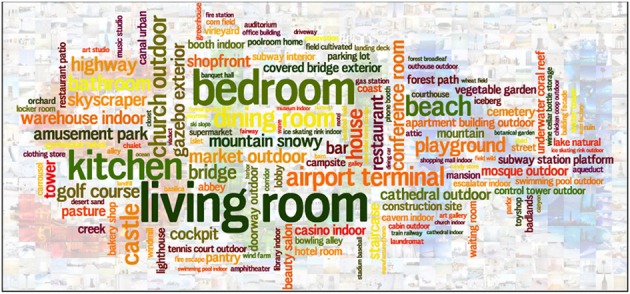
**List of 908 scene categories in our SUN database—the most exhaustive scene dataset to date**. The height of each category name is proportional to the number of images belonging to the category.

To capture this diversity, we have constructed a quasi-exhaustive taxonomy and dataset of visual scene categories that can be encountered in the world. We use WordNet, an electronic dictionary of the English language containing more than 100,000 words, and manually select all of the terms that describe scenes, places, and environments (any concrete noun that could reasonably complete the phrase “I am in a *place*”, or “Let's go to the *place*”). This has yielded 908 scene categories, which are illustrated in Figure 5.

Once we have a list of scenes, the next task is to collect images belonging to each scene category. Since one of our goals is to create a large collection of images with variability in visual appearance, we have collected Internet images using various image search engines for each scene category term. Then, a group of trained human participants manually prune the images that do not correspond to the definition of the scene category resulting in a database of 131,072 images. This collection of images is the core of the SUN database onto which all other annotations discussed are added. Using a variety standard image features (e.g., spatial pyramids of dense visual words) one can achieve roughly 40% accuracy in a 397-way scene categorization task (Xiao et al., 2010). Recent work has achieved 47% accuracy (Sanchez et al., 2013). We have also studied intra-category variations in the SUN database. Within the same scene category, human observers find some exemplars to be more typical than others and category membership is naturally graded, not discrete (Ehinger et al., 2011).

## 3. Scene attributes

In this section we present the SUN attribute database—the first large-scale scene attribute database (Patterson and Hays, 2012). Recently, there has been interest in *attribute-based* representations of objects (Farhadi et al., 2009; Lampert et al., 2009; Berg et al., 2010; Endres et al., 2010; Farhadi et al., 2010; Russakovsky and Fei-Fei, 2010; Su et al., 2010), faces (Kumar et al., 2009), and actions (Liu et al., 2011; Yao et al., 2011) as an alternative or complement to category-based representations. However, there has been only limited exploration of attribute-based representations for scenes (Oliva and Torralba, 2001, 2002; Greene and Oliva, 2009; Parikh and Grauman, 2011), *even though scenes are uniquely poorly served by categorical representations*. For example, an object usually has unambiguous membership in one category. One rarely observes objects at the transition point between object categories (e.g., this object is on the boundary between “sheep” and “horse”), however, the analogous situation is common with scenes (e.g., this scene is on the boundary between “savanna” and “forest”).

In the domain of scenes, an attribute-based representation might describe a image with “concrete,” “shopping,” “natural lighting,” “glossy,” and “stressful” in contrast to a categorical label such as “store”. Note that attributes do not follow category boundaries. Indeed, that is one of the appeals of attributes—they can describe intra-class variation (e.g., a canyon might have water or it might not) and inter-class relationships (e.g., both a canyon and a beach could have water). We limit ourselves to *global, binary* attributes. but we average the binary labels from multiple annotators to produce real-valued confidences.

**Figure 6:**
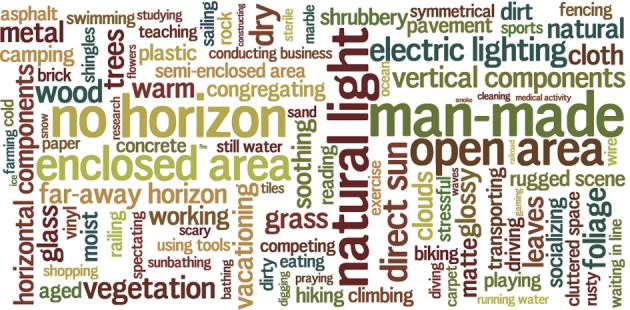
**Attributes in the SUN Attribute database**. The area of each word is proportional to the frequency of that attribute.

Our first task is to establish a taxonomy of scene attributes for further study. We use a simple, crowd-sourced “splitting task” (Oliva and Torralba, 2001) in which we show Amazon Mechanical Turk (AMT) workers two groups of scenes and ask them to list attributes that are present in one group but not the other. The images that make up these groups are “typical” (Ehinger et al., 2011) scenes from random categories of the SUN database. From the thousands of attributes reported by participants we manually collapse nearly synonymous responses (e.g., dirt and soil) into single attributes. We omit object presence attributes because the SUN database already has dense object labels for many scenes. In the end, we arrive at a taxonomy of 38 material attributes (e.g., cement, vegetation), 11 surface properties (e.g., rusty), 36 functions or affordances (e.g., playing, cooking), and 17 spatial envelope attributes (e.g., enclosed, symmetric). See Figure 6 for the full list.

With our taxonomy of attributes finalized, we create the first large-scale database of attribute-labeled scenes. We build the SUN attribute database on top of the existing SUN categorical database (section 2) for two reasons: (1) to study the interplay between attribute-based and category-based representations and (2) to ensure a diversity of scenes. We annotate 20 scenes from each of 717 SUN categories totaling 14,340 images. We collect ground truth annotations for all of the 102 attributes for each scene. In total we gather more than four million labels through crowdsourcing. After labeling the entire dataset once with the general AMT population, we identify a smaller group of 38 trusted workers out of the ~800 who participated. We repeat the labeling process two more times using only these trusted workers.

### 3.1. Building the sun attribute database

To quantitatively assess annotation reliability we manually grade random annotations in the database. Ninety-three percent positive annotations are reasonable (some are undoubtedly subjective). The negative annotations also have 93% accuracy, but this isn't as significant since negative labels make up 92% of the annotations. Like objects, it seems that scene attributes follow a heavy-tailed distribution with a few being very common (e.g., “natural”) and most being rare (e.g., “wire”). If we instead evaluate the consensus annotation which two of the three annotators agree on for each scene attribute, the accuracy rises to 95%.

#### 3.1.1. Exploring scenes in attribute space

**Figure 7:**
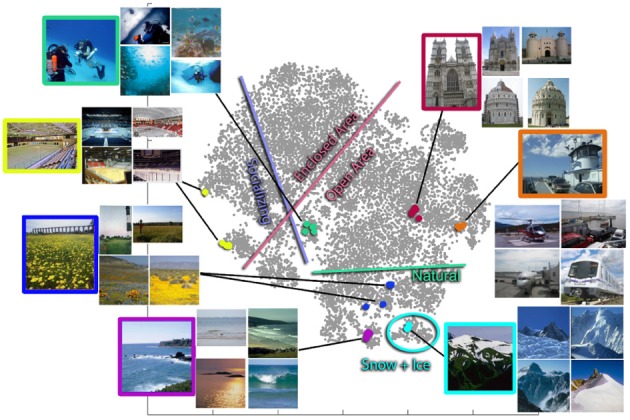
**2D visualization of the SUN Attribute dataset**. Each image in the dataset is represented by the projection of its 102-dimensional attribute feature vector onto two dimensions using t-Distributed Stochastic Neighbor Embedding. There are groups of nearest neighbors, each designated by a color. Interestingly, while the nearest-neighbor scenes in attribute space are semantically very similar, for most of these examples (underwater ocean, abbey, coast, ice skating rink, field wild, bistro, office) *none* of the nearest neighbors actually fall in the same SUN database category. The colored border lines delineate the approximate boundaries between images with and without the particular attributes.

**Figure 8:**
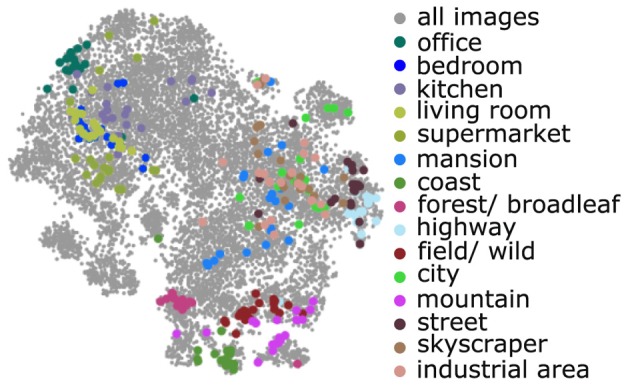
**The 15 scene category database images in attribute space**. These 15 proxy categories occupy a relatively small fraction of attribute space spanned by the SUN database (all gray points).

Now that we have a database of attribute-labeled scenes we can attempt to visualize that space of attributes. In Figure 7 we show all 14,340 of our scenes projected onto two dimensions by dimensionality reduction. We sample several points in this space to show the types of scenes present as well as the nearest neighbors to those scenes in attribute space. For this analysis the distance between scenes is simply the Euclidean distance between their real-valued, 102-dimensional attribute vectors. Figure 8 shows the distribution of images from 15 scene categories in attribute space. The particular scene categories were chosen to be close to those categories in the 15 scene database (Lazebnik et al., 2006).

### 3.2. Recognizing scene attributes

**Figure 9:**
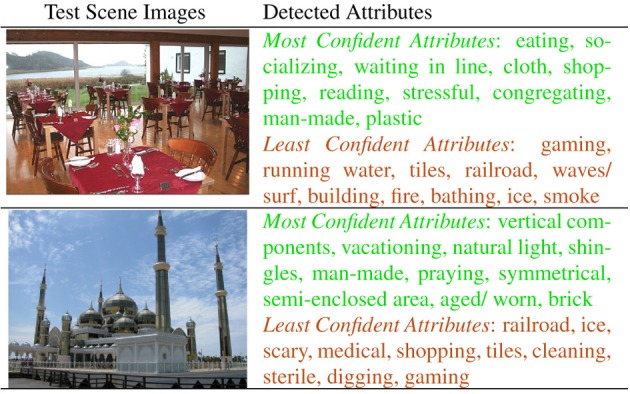
**Attribute detection**. For each query, the most confidently recognized attributes (green) and the least confidently recognized attributes (red).

To recognize attributes in images, we create an independent classifier for each attribute using splits of the SUN Attribute dataset for training and testing data. We treat an attribute as present if it receives at least two of three possible votes from AMT annotators and absent if it receives zero votes. We represent each image with a subset of the features and kernels used for scene categorization in Xiao et al. (2010). We train Support Vector Machines on 90% of the SUN Attribute dataset and test on the remaining 10%. Figure 9 shows the attributes detected in two query scenes. Attribute recognition accuracy varies considerably, e.g., average precision of 0.93 for “vegetation,” 0.78 for “sailing,” 0.60 for “moist,” and 0.27 for “stressful.” We show qualitative results of our attribute classifiers in Figure 9. Our classifiers and the code are publicly available^1^.

## 4. Scene structures

Although an image is a 2D array, we live in a 3D world, where scenes have volume, affordances, and can be spatially arranged where one object can be occluded by another. The ability to reason about these 3D properties would be of benefit for tasks such as navigation and object manipulation.

**Figure 10:**
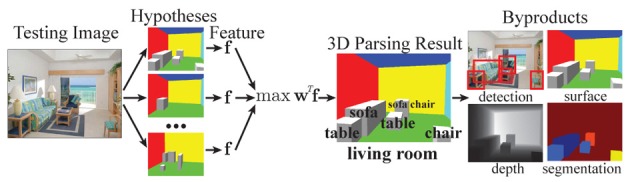
**Unified 3D scene parsing for basic level scene understanding**. For each image, we generate a pool of hypotheses. For each hypothesis, we construct a feature vector **f** encoding both image features and scores from evaluating various contextual rules on the hypothesized scene structure (Figure 11). We choose the hypothesis that maximizes the objective function **w**^*T*^**f** as the result of 3D scene parsing. As by-products of our 3D parsing result we obtain information that have traditionally been considered in isolation, such as the 2D location of objects, their depth and 3D surface orientation.

We seek to build a unified framework for parsing the 3D structure of a scene. What does it mean to parse a scene? There is no universal answer, as it heavily depends on the tasks (e.g., the task of a house painter is to find all cracks on a wall). Here, we limit our scope to the basic 3D properties of the space, including the scene category, the 3D boundary of the space, and all the objects in the image. For each object, we want to know its category and 3D bounding box, including its orientation. As an image is a viewer-centric observation of the space, we also want to recover the camera intrinsic and extrinsic parameters. An example 3D parse result is depicted in Figure 10 for a living room scene.

**Figure 11:**
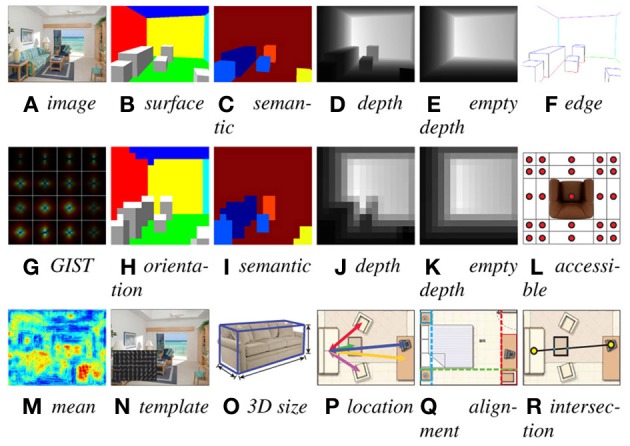
**(A–R)** Illustration of various rules we design to describe both the image evidence and context compatibility. All these rules are encoded in the structural SVM feature function **f**(**x**, **y**).

While it is possible to reason about these various scene properties independently, we desire an algorithm which considers them jointly. Thus an algorithm might suppress a false positive “bed” detection because it is sitting on a “table”. There are numerous such scene layout “rules” which constrain the parsing of a scene and optimizing a scene parsing with respect to all such rules could lead to complicated inference procedures. The key idea of our algorithm is to generate a pool of possible output hypotheses and select the most likely one. We define a list of parsing rules and use structural Support Vector Machine (SVM) (Joachims et al., 2009) to learn the relative importance of these rules from the training data. For each hypothesis, we extract a vector which encodes how well each rule is satisfied by the hypothesis. The weight of each element in this vector in scoring the hypotheses is learned from training data. More specifically, given an image **x**, we aim to predict a structured representation **y** for the 3D parsing result using a linear prediction rule: 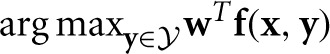, where 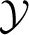 is the hypothesis space of all possible 3D parsing results for **x**. The label **y** is a variable dimension data structure of a 3D scene parsing, which includes the scene category, camera parameters, space boundaries, and objects^2^. We encode image evidence and contextual constraints into the feature vector **f**(**x**,**y**). Therefore, a good scene parsing result **y** not only explains the image evidence well, but also satisfies the contextual constraints. The parsing rules are illustrated in Figure 11.

During training, given a training sample of input–output pairs ((**x**_1_,**y**_1_),…,(**x**_*N*_, **y**_*N*_)) from manual annotation (we add annotations to the data set of Hedau et al., 2012), we seek to minimize the following convex optimization problem:

(1) $$\min\frac{1}{2}w^{T}w+C\sum_{n=1}^{N}\xi_{n},$$

such that **w**^*T*^**f**(**x**_*n*_, **y**_*n*_) − **w**^*T*^**f**(**x**_*n*_, **ŷ**) ≥ △(**y**_*n*_, **ŷ**) − ξ_*n*_, for all 1 ≤ *n* ≤ *N* and for all possible output structures 
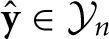
 in the hypothesis space. △(**y**_*n*_, **ŷ**) is the loss function controlling the margin between correct label **y**_*n*_ and prediction **ŷ**^3^.

One of the major differences between structural SVM and standard SVM is that the feature **f**(**x**, **y**) depends not only on **x**, but also on **y**. This enables us to encode many important image features and context rules (section 5) that were not possible in previous works. Moreover, the SVM discriminatively learns the relative importance of features and relations based on training data. For training, we use the cutting plane algorithm (Joachims et al., 2009; Desai et al., 2011). For each training image **x**_*n*_, we use simple heuristics to obtain a hypothesis pool 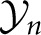 (section 6), to be the initial working constraints to train the SVM. As the training goes on, we add more hypotheses with large **w**^*T*^**f** values as working constraints, based on the current **w**. It can be seen as a generalization of hard negative mining in sliding window object detection (Dalal and Triggs, 2005; Felzenszwalb et al., 2010), and it significantly speeds up the computation and reduces memory consumption.

### 4.1. Related work

Coughlan and Yuille (1999); Delage et al. (2005); Hoiem (2007); Saxena et al. (2009): reconstruct surface orientation and depth from single view images. Han and Zhu (2005); Yu et al. (2008); Hedau et al. (2009, 2010); Lee et al. (2009, 2010); Wang et al. (2010); Gupta et al. (2011); Pero et al. (2011, 2012); Yu et al. (2011); Zhao and chun Zhu (2011); Hedau et al. (2012); Schwing et al. (2012): represent the state-of-the-art on room layout estimation and furniture arrangement. There are also many impressive techniques to model context and object relations (Hoiem, 2007; Rabinovich et al., 2007; Desai et al., 2011), and parse a scene (Han and Zhu, 2005; Heitz et al., 2008; Socher et al., 2011; Li et al., 2012) in a unified way for multiple tasks at the same time. Although they have some success on reasoning about 3D, their main focus is still on 2D. Meanwhile, structural SVMs (Joachims et al., 2009) have been successfully applied to many computer vision tasks (Hedau et al., 2009; Felzenszwalb et al., 2010; Wang et al., 2010; Desai et al., 2011; Gupta et al., 2011; Schwing et al., 2012). The main difference is that these approaches have not learned or predicted as rich and complex structures as ours.

## 5. Parsing rules

A good scene parsing result **y** not only explains the image evidence well, but also satisfies contextual constraints. Inspired by natural language parsing (NLP) using structural SVM (Joachims et al., 2009), we encode both types of rules—image likelihood and context constraints—into the feature vector **f**(**x**,**y**). The structural SVM automatically figures out which rules are important discriminatively based on the training data. The image likelihood rule is evidence of the form “This set of pixels looks like a bed” while higher-order, contextual rules are of the form “a bed is in a bedroom”. We accumulate all rules being used in a unified way into one fixed length **f**, for both image and context rules defined below. The scoring function **w**^*T*^**f**(**x**, **y**) for a hypothesis **y** is essentially a weighted sum of the scores from these rules.

### 5.1. Region features

Given **y**, the surface orientation is fully determined for each pixel, as shown in Figure 11B. We define several categories of surface orientation, including floor, ceiling, left, right, and frontal-parallel walls, as well as top, bottom, left, right, and frontal-parallel faces of a 3D object bounding box. For each image **x**_*n*_, we precompute a pixel-wise image feature map **F**_*n*_ (Figure 11M), using several state-of-the-art image features—SIFT (Lowe, 2004), 3 × 3 HOG template centered at each pixel (Dalal and Triggs, 2005; Felzenszwalb et al., 2010), Self-Similarity (Shechtman and Irani, 2007), and distance transformed Canny edge map, as well as the pixel locations, for a total of 440 dimensions. Figure 11M visualizes the mean feature values. To form a feature vector, for each surface orientation category, we aggregate **F**_*n*_ to sum the feature vectors over all pixels with this orientation category, based on the orientation map. For example, we sum over all feature vectors belonging to the floor by ∑_(*x, y*)∈floor_**F**_*n*_(*x, y*,:) to obtain the feature vector for the floor. Similar to surface orientation, we determine the semantic segmentation map using 3D bounding boxes and aggregate the image features to make another set of features based on object categories. In Figure 11C, different colors correspond to different object categories. Furthermore, inspired by scene classification, to encode the global structure of the scene, we extract the GIST feature (Oliva and Torralba, 2001) for the whole image, as shown in Figure 11G.

### 5.2. Edge and corner features

We extract edges of the space boundaries and object bounding boxes, and aggregate the image feature over edges. As shown in Figure 11F, we further separate the edges into six categories, including ground line, ceiling line, vertical wall edges as well as the top, bottom, and vertical edges of an object bounding box. For each type, we aggregate the image feature in the same way as the region features. In a similar way, we also extract corner features, for four types of corners: floor corners, ceiling corners, as well as the top and bottom of objects.

### 5.3. Holistic segmentation and depth statistics

As shown in Figure 11H, to encode the global layout of surface orientation, we extract holistic statistics of the map. We down-sample the full resolution surface orientation map in Figure 11B in a spatial pyramid of 16 × 16, 8 × 8, 4 × 4, 2 × 2, and 1 × 1 resolutions, and concatenate all of the values together to form a holistic feature of surface orientation. We do the same for semantic segmentation as well to encode the holistic statistics of the semantic map (Figure 11I). Furthermore, given **y**, the depth of the scene is fully determined, which is a strong contextual criterion to judge whether a parsing result is possible independent of the image observation. We can obtain the depth map (Figure 11D), and extract a holistic depth feature with spatial pyramid (Figure 11J). We also empty the room to extract the depth map of the room structure (Figure 11D), and build the spatial pyramid as well (Figure 11K). We compute the average depth map for each scene category, and use the difference between the current depth and the mean depth as part of features. The depth encodes some statistics of typical views, e.g., a camera looking directly at a nearby wall is not likely to be a good hypothesis.

### 5.4. Object 2D templates, color and texture consistency

Inspired by sliding window object detection, for each object, we obtain the 2D bounding box from the 3D bounding box, and compute a HOG template (Dalal and Triggs, 2005; Felzenszwalb et al., 2010) of the 2D window (Figure 11N), as a view-dependent object template feature. For each object category, we obtain the average aspect ratio of the ground truth from the training data, and adjust the individual instances to have the same aspect ratio and template resolution during feature extraction.

### 5.5. Object 3D size and location

Different object categories have very different absolute sizes and locations. Therefore, we extract the 3D size as features (Figure 11O). For the location of an object, because it is usually relative the space boundary, e.g., a bed flush to the wall, we find the closest vertical space boundary for an object, and use their distance and angle difference as features.

### 5.6. Human accessibility

For the arrangement of objects, there is usually some space between objects for them to be accessible by humans. For example, we tend to have some space in front of a sofa to enable people to walk and sit there. To encode this information as a feature vector, as shown in Figure 11L, we put a 5 × 5 horizontal spatial grid that is 2 feet away around an object. We would like to record whether some space in each bin is occupied by other objects or outside the room. Because this computation is very expensive, we instead use only the center location of each bin, and check if it is inside some other objects or outside the room, to approximate the accessibility. We concatenate the occupancy information together to form a feature vector.

### 5.7. Co-occurrence and relative location

Given **y**, we obtain the object co-occurrence count for each pair of object categories as our co-occurrence context feature. We also count how many times an object appears together with the scene category. For both types of occurrence relationship, we obtain the average statistics of the training data, and use the difference between the current statistics and the average one as features. Beyond co-occurrence, objects usually have certain position constraints with each other. For example, a sofa is usually parallel with a coffee table with a certain distance. Therefore, we encode the relative 3D distance and orientation difference of their bounding box as features (Figure 11P). Also, many objects tend to be arranged so that certain faces are aligned (Figure 11Q). Therefore, we encode the pairwise alignment relationship between objects, by checking whether certain facets are parallel or very close in the space. This is not as strong as imposing the Manhattan world assumption, but it encourages snapping of edges and corners.

### 5.8. Higher order relationships

Object relation is not just pairwise. For example, in a typical living room, between a sofa and a TV, there may be a coffee table but it is unlikely to be a tall object blocking the view between them. To encode this high order relationship, for each pair of objects, we draw a line segment connecting them, and check whether any other objects have bounding boxes intersecting this line segment (Figure 11R). If yes, we will record the object category and use this as a feature.

### 5.9. Camera and view constraints

When people take a picture, the camera is not randomly rotated and the intrinsic parameters must be in certain range constrained by the camera hardware. Therefore, we can encode the camera parameters as features as well. Also, we want to represent the volume of visual space in the field of view. To simplify the computation, we reconstruct the floor in 3D and calculate the 3D area of that part that is visible from the camera. We use the 3D area, and the difference of the area to the average floor area across the training set as a feature.

For all these rules, some of them are unary features that encode rules about one instance of the object, and some of them are pairwise or higher order relationship among objects and scenes. Some rules are hypothesis independent features in the sense that their values do not change because of **y**, while others are hypothesis dependent features with values depending on **y**. Some features are view-independent and encode the absolute 3D statistics independent of the camera, while others are view dependent features that heavily depend on the camera.

## 6. Hypothesis generation

**Figure 12:**
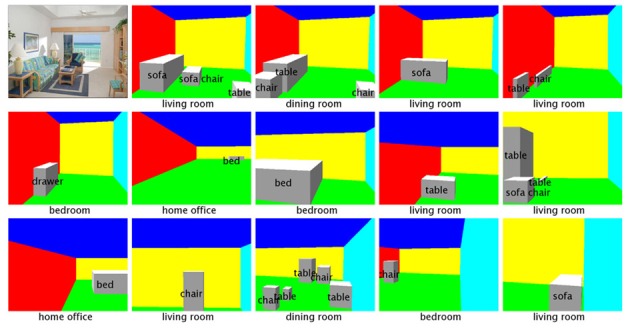
**Random subset of hypotheses generated for one image, ordered increasingly by their values of loss function △(y_*n*_, ŷ)**.

We propose a two step algorithm for generating hypotheses and performing fast inference. For any image, either during training or testing, we first generate a large pool of initial hypotheses, without considering the objective function. Then, we do several iterations of heuristic search, based on the initial hypothesis pool and **w**, and simply pick the one with the highest objective value **w**^*T*^**f** as the solution. Figure 12 shows some examples of hypotheses generated.

For the first step of constructing an initial hypothesis set, we use two approaches. First, we use a bottom–up approach to generate some hypotheses based on image evidence. We detect edges and fit line segments, to obtain camera parameters from vanishing points at orthogonal directions. To look for reliable line segments and vanishing points, we use many state-of-the-art line detection algorithms (Han and Zhu, 2005; Toldo and Fusiello, 2008; Hedau et al., 2009; Tardif, 2009; Feng et al., 2010; Lee et al., 2010; von Gioi et al., 2012). For each set of camera parameters estimated from all these methods, if the focal length is outside the range of a normal digital camera (we use 24–105 mm), or the principal point is far away from the center of the image, we remove the estimated camera from further considerations. In this way, we usually obtain about seven good camera models per image, out of hundreds estimated by all methods. For each of the cameras, we randomly sample some edges to form a hypothesis for the space boundary and group line segments into cuboids (Lee et al., 2010). We also exhaustively slide cuboids in 3D for each object category using the average size of objects in the category from the training set. We explicitly enforce the support relationship to make sure each objects are grounded on the floor or on top of other objects. The second way to generate hypotheses is to copy ground truth hypotheses from the training set and add them into the initial hypothesis space. We also generate hypotheses by using the estimated cameras from line detection and replace them in the copied training set labels. With all these hypotheses, we randomly sample a subset of all hypotheses as our initial hypothesis pool. For the training data, we also randomly change the ground truth to generate more training hypotheses nearby the truth to learn a better **w**.

The second step is to obtain some better hypotheses based on **w** by modifying the initial hypotheses. This is used to find the most violated constraints during training, and a best solution during testing. We use **w** to pick 200 top hypotheses and randomly choose another 200 hypothesis as our working set. For each of these 400 hypotheses, we adjust them in various ways to generate new hypothesis, in a randomized fashion. We allow the algorithm to change the space boundary positions, scene category, all object properties including category, location, rotation and size. We also allow removing some objects, or adding some objects. We iteratively run this step for several times, until we cannot find any better solutions in two consecutive iterations.

**Figure 13:**
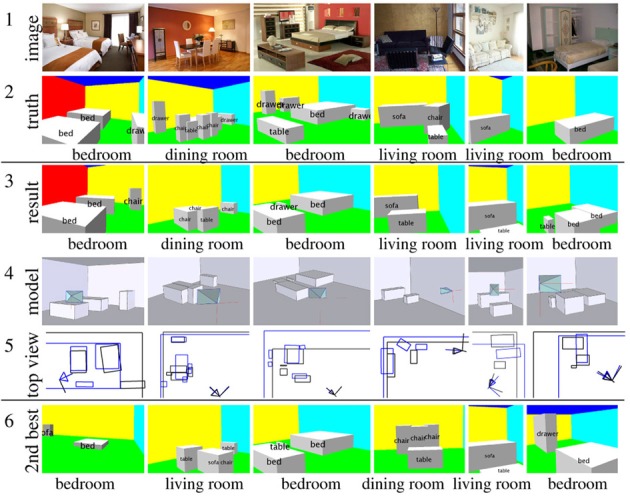
**Example results of our algorithm**. The 1st row contains the input test image. The 2nd row contains the ground truth. The 3rd row contains a 2D rendering of our 3D parsing results. The 4th row is the rendering of the 3D mesh model and the camera frustum from a different viewpoint. The 5th row is the top view of the 3D parsing result: the 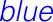 lines are the ground 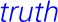, and the *black* lines are the *result*. The last row is the 2nd best prediction, which gives us an alternative parsing result.

**Figure 14:**
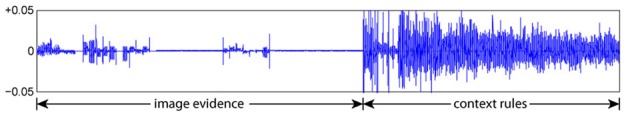
**Visualization of the w learned by the structural SVM**. We group the weights into two separate categories roughly corresponding to image evidence and context constraints. We can see that the model puts larger weights on context features, which indicatives that they are discriminatively more informative. Note that we handle feature normalization carefully so that different features are at similar order of magnitude.

We show example outputs in Figure 13. Notice that our algorithm correctly detects many objects and recovers the 3D layout of a variety of scenes. In the first column, although our model fails to recognize the drawers, it manages to recognize a chair that is not labeled in the ground truth due to labeling error. In column 5, row 3 of Figure 13 we see that our model mistakes the white carpet in front of the sofa as a table, which is a typical configuration for a living room. We also visualize the actual learned weights in Figure 14. This shows that the contextual cues are found to be very important for evaluating hypotheses.

## 7. Conclusion

We have proposed basic level scene understanding as a tractable research goal, and have summarized our recent effort to probe the state of the art of several domains and questions related to visual scene understanding. First, we describe the richly annotated SUN database with object, attribute, and geometric labels for many scenes. This database allows us to systematically study the space of scenes and to establish a benchmark for scene and object recognition. Furthermore, we augment the categorical SUN database with millions of scene attribute labels and explore attribute recognition. Finally, we propose a unified framework for parsing a 3D scene to generate a basic level 3D representation. By modeling the task as a structural SVM problem, we train a complete end-to-end system in a single step by optimizing a unified objective function. With our proposed image and context rules, the SVM automatically weighs the relative importance of the rules based on training data. Current and future investigations are concerned with applications of the work to domains, such as image-based modeling (Xiao et al., 2008, 2009; Xiao and Quan, 2009; Xiao and Furukawa, 2012), viewpoint extrapolation (Xiao et al., 2012; Zhang et al., 2013), and assessment of subjective visual scene properties (Isola et al., 2011; Khosla et al., 2012a,b).

### Conflict of interest statement

The authors declare that the research was conducted in the absence of any commercial or financial relationships that could be construed as a potential conflict of interest.

## Footnotes

^1^SUN Attribute Classifiers along with the full SUN Attribute dataset and associated code are available at www.cs.brown.edu/~gen/sunattributes.html.

^2^We assign the origin of the world coordinate system to be at the camera location, and set the gravity direction as the negative *y* axis. The unknown camera parameters are focal length, principal point and the camera rotation. The space is parameterized as [*x*_min_, *x*_max_]× [*y*_min_, *y*_max_] × [*z*_min_, *z*_max_], with the assumption that the space boundaries, e.g., walls, floor and ceiling, are perpendicular to each other. The objects in the scene are represented as a set of objects grounded on the floor or stacked on another object. Each object is represented as an object category, a 3D bounding box, including its center location (*x*_*c*_, *y*_*c*_, *z*_*c*_), its size (*x*_*s*_, *y*_*s*_, *z*_*s*_), and its yaw angle θ, with the assumption that the vertical axis of the bounding box must be parallel with the gravity direction.

^3^To measure the 2D observation difference, between **y**_*n*_ and **ŷ**, we render both 3D meshes to obtain two surface orientation maps. We compare the two maps at each pixel and obtain the accuracy Ω_*g*_ over all pixels. We do the same thing for semantic segmentation masks to obtain the pixel-wise accuracy Ω_*s*_. Because 2D errors are sometimes not very indicative of actual 3D errors, we also measure the object correctness in 3D and define Ω_*o*_ as the proportion of overlapping objects which share the same category. Finally, we measure the scene category correctness Ω_*h*_. The weighted sum of all these measurement is our total loss function: △(**y**_*n*_,**ŷ**) = *w*_*g*_ (1 − Ω_*g*_) + *w*_*s*_ (1 − Ω_*s*_) + *w*_*o*_ (1 − max(Ω_*o*_, 0)) + *w*_*h*_(1 − Ω_*h*_).
